# Physical Activity Reduces the Risk of Fragility Fracture

**DOI:** 10.1371/journal.pmed.0040222

**Published:** 2007-06-19

**Authors:** Harri Sievänen, Pekka Kannus

## Abstract

The authors discuss a new study, with a 35 year follow up, showing that exercise reduces the risk of fragility fractures in men.

From an evolutionary perspective, we were born to run [1]. Indeed, the human musculoskeletal system is a fine locomotive apparatus that can adapt to varying demands. Our bones and muscles have the inborn ability to modify their structural and material designs to accommodate additional loads. Present-day athletes, for example, show higher bone mass and more robust structure than nonathletes at loaded bone sites [2].

Studies of skeletal remains show skeletal strength has fallen relative to body size over the course of human evolution, which is thought to be due to the rise in sedentary lifestyles [3]. The loss of bone strength associated with the typical sedentary lifestyles of people in the developed world may result in a higher risk of fractures. One recent study, for example, comparing the proximal femur of medieval and contemporary adults, found that over about 1,000 years the femoral neck axis has become longer and its cross-section has become proportionally smaller [4]. This change in the phenotype of the proximal femur has led to a 50% increase in fall-induced stress upon the bone compared with medieval times [4].

Obviously, a weak bone is more likely to fail than a strong one, but without the excess stress caused by falls, even a fragile bone may cope well with normal living [5]. The root cause of most fragility fractures is falling, which leads to a large load on the bone from a direction it is not particularly adapted to handling. Fractures due to falls among aging people have become a serious public health problem for our modern societies [6,7], so there is great interest in finding preventive strategies. Regular exercise is undoubtedly one of the most promising preventive options, particularly because of its proven benefits for both bone rigidity and neuromuscular performance [7].

**Linked Research Article**

This Perspective discusses the following new study published in *PLoS Medicine:*

Michaëlsson K, Olofsson H, Jensevik K, Larsson S, Mallmin H, et al. (2007) Leisure physical activity and the risk of fracture in men. PLoS Med 4(6): e199. doi:10.1371/journal.pmed.0040199

From a large cohort study with 35 years of follow-up, Michaëlsson and colleagues conclude that regular sport activities can reduce the risk of fractures in older men.

## New Research on Exercise and Fractures

The best way to prove that physical activity prevents fragility fractures would be to conduct several adequately powered randomized controlled trials. But such trials are almost impossible to do for methodological reasons, such as problems with study size, compliance, drop-outs, blinding, and long-term follow-up [8]. But there is evidence from several prospective observational cohort studies that physical activity is associated with a reduced risk of fragility fracture [9–12]. A 2002 study of postmenopausal women by Feskanich et al. [13] found a dose-response relationship: the risk of hip fracture was lowered by 6% for each increase of three metabolic equivalent hours of activity per week (equivalent to one hour per week of walking at an average speed).

The evidence from such cohort studies in men, however, has been inconclusive—some studies have reported significant reductions in risk of fragility fracture with physical activity but others found no such reduction (the studies are listed in [8]). Given these inconsistent findings, the results of a new study published in *PLoS Medicine* by Karl Michaëlsson and colleagues, involving a cohort of 2,205 men followed for 35 years, represent an important advance [8].

The researchers classified the men into three groups according to whether their level of physical activity was high, medium, or low. At the end of the follow-up, 8.4% of the men in the high physical activity group, 13.3% of the men in the medium physical activity group, and 20.5% of the men in the low physical activity group had suffered a hip fracture.

In Michaëlsson and colleagues' study [8], the effective “dose” of exercise for reducing the risk of hip fracture was readily attained by recreational sports, heavy gardening, or other activities with similar intensity performed for at least three hours per week. This volume of exercise is relatively easy to incorporate into everyday life. It is also fortunate that it is never too late to start exercising. Even during adult years, initiation or increase of regular physical activity can reduce the risk of fracture [8,13]. The greatest challenge is to get both younger and older people moving and to keep them active and avoid sedentary lifestyles [14].

## Implications for Policy and Future Research

Fractures can always happen, but the two studies by Feskanich et al. [13] and Michaëlsson et al. [8] show that the risk of fragility fractures can be reduced by regular physical activity. According to Michaëlsson and colleagues' estimation of population-attributable risk, one-third of all hip fractures could be avoided if men engaged in adequate amounts of physical activity [8], a result that is fully concordant with a similar analysis in women [12]. These data cannot be ignored by public health professionals and policymakers. Substantial efforts should be put into promoting physical activity in the population at large.

Although the case has now been made that physical activity helps to prevent fractures, there are still some unsolved questions regarding the type, intensity, frequency, and duration of activity that would constitute the optimal exercise regimen in different target groups. So far, the description of exercise regimens intended for strengthening the skeleton and improving physical performance have been superficial and based, at least partly, on subjective assessments of a person's habitual activity only. However, without quantitative, reliable information on the exercise stimulus and response, the precise dose–response relationship will remain elusive. Many of the outcome variables reported in the existing exercise studies, such as areal bone mineral density or isometric muscle force, do not provide sufficient information to determine such dose–response relationships.

Future clinical intervention trials investigating the effects of exercise on fragility fractures and their risk factors should be based on (1) reliable assessments of whole-bone structure; (2) measurements of exercise-induced loading, muscle performance, and balance in real-life situations; and (3) collection of data on falls and fractures.
